# Advances and Challenges in the Diagnosis of Vector-Borne Protozoal Infections in Veterinary Medicine

**DOI:** 10.3390/pathogens15060561

**Published:** 2026-05-22

**Authors:** Ana María Cevallos, Tomas Meraz-Tay, Roberto Hernández

**Affiliations:** Departamento de Biología Molecular y Biotecnología, Instituto de Investigaciones Biomédicas, Universidad Nacional Autónoma de México, Ciudad de México 04510, Mexico; tomasmeraztay@gmail.com (T.M.-T.); robertohf@biomedicas.unam.mx (R.H.)

**Keywords:** *Babesia*, blood smear, LAMP, *Leishmania*, One Health, PCR, serology, surveillance, *Theileria*, *Trypanosoma*, vector-borne protozoa, veterinary diagnostics

## Abstract

Vector-borne protozoal infections—including babesiosis, theileriosis, hepatozoonosis, trypanosomosis, and leishmaniosis—impose a substantial burden on livestock and companion animal health worldwide and carry important zoonotic and public health implications. Accurate diagnosis is essential yet challenging, given the diversity of parasite genera, their markedly different tissue tropisms, and the uneven distribution of diagnostic resources across veterinary settings. This review provides an integrated overview of the principal diagnostic approaches available, structured around the biological logic that guides test selection in practice. Microscopic examination remains the first-line method; its strengths and limitations are discussed for intraerythrocytic parasites (*Plasmodium* spp., *Babesia* spp., *Theileria* spp., *Cytauxzoon* spp.—the latter two with additional extra-erythrocytic schizont stages in leukocytes and tissue macrophages, respectively), leukocyte-associated forms (*Hepatozoon* spp.), extracellular trypanosomes, and tissue-stage parasites, including emerging applications of artificial intelligence. Serological methods—enzyme-linked immunosorbent assay (ELISA), indirect fluorescence antibody test (IFAT), and point-of-care lateral flow assays—are evaluated for their role in exposure detection, population screening, and international trade certification, with attention to cross-reactivity and the active-versus-past-infection distinction. Molecular diagnostics, encompassing conventional PCR, qPCR, droplet digital PCR, isothermal amplification, and next-generation sequencing, are reviewed with respect to target selection, sensitivity, and point-of-care applicability. Finally, diagnostic challenges are contextualised within a One Health framework, highlighting the fragmentation of veterinary surveillance and the need for integrated, cross-sector approaches to detect emerging threats.

## 1. Introduction

Protozoal infections transmitted by arthropod vectors such as ticks, flies, and mosquitoes—including babesiosis, theileriosis, hepatozoonosis, trypanosomosis, and leishmaniosis—affect both livestock and companion animals worldwide, causing significant levels of morbidity and mortality. Beyond their direct impact on animal welfare, these infections impose a substantial economic burden, reducing livestock productivity through decreased milk yield, weight loss, reproductive failure, and mortality, and generating considerable costs associated with treatment, prevention, and vector control programmes. Several of these parasites are also zoonotic or have wildlife reservoir hosts that place them at the human–animal–ecosystem interface, making their accurate diagnosis a priority not only for individual animal health but also for public health surveillance.

**Figure 1:**
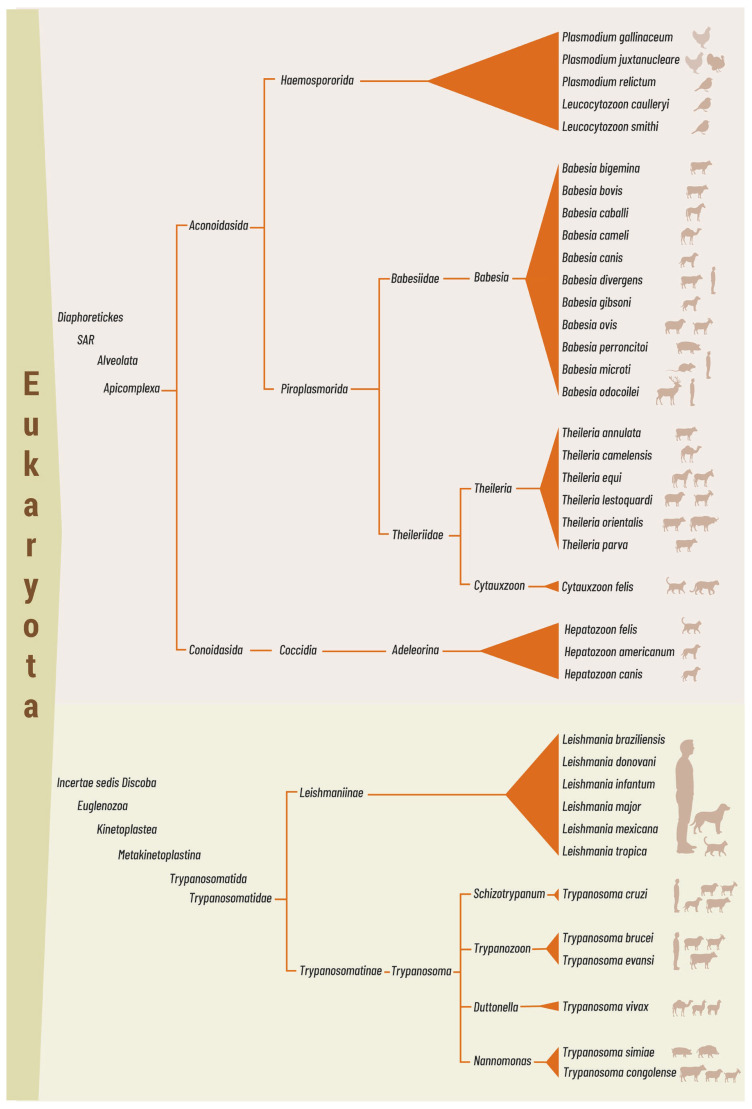
Taxonomic distribution of vector-borne protozoa of veterinary importance and their vertebrate hosts (indicated by silhouettes). The classification shown integrates two complementary frameworks. Higher-level clades—Diaphoretickes, SAR, Alveolata, Discoba, and Euglenozoa—follow Adl et al. [1], who propose them as a hierarchy of nested clades without formal Linnaean rank designations. Linnaean ranks (class, order, family, subfamily, genus, subgenus) follow the NCBI Taxonomy Browser, as Adl et al. [1] only informally suggest phylum- and class-level positions and do not formalise lower ranks. The placement of Discoba within the eukaryotic tree remains unresolved and is therefore indicated as *incertae sedis*; in contrast, Apicomplexa is securely nested within the SAR clade of Diaphoretickes. Within the family Trypanosomatidae, the vector-borne genera shown belong to two subfamilies: Leishmaniinae (containing *Leishmania*) and Trypanosomatinae (containing *Trypanosoma*).

Among protozoa, vector-borne parasites—those that require an arthropod for biological development as part of a two-host life cycle—have evolved within a limited number of specific lineages across two distinct eukaryotic groups: the informal clade Discoba (*incertae sedis*) and the domain Diaphoretickes (Adl et al. [1]). Within Discoba, the relevant lineages belong to the order Trypanosomatida, family Trypanosomatidae; among the subfamilies of this family, the vector-borne genera fall into two: Leishmaniinae (genus *Leishmania*) and Trypanosomatinae (genus *Trypanosoma*). Among the seven subgenera of *Trypanosoma*, four contain vector-borne species of veterinary importance: *Schizotrypanum* (e.g., *Trypanosoma cruzi*), *Trypanozoon* (e.g., *Trypanosoma brucei*), *Duttonella* (*Trypanosoma vivax*), and *Nannomonas* (*Trypanosoma simiae*, *Trypanosoma congolense*) (Figure 1). Beyond their taxonomic placement, the *Trypanosoma* subgenera differ in features that have direct clinical and management implications. Most notably, they are transmitted by different routes: *Schizotrypanum* parasites are stercorarian, transmitted through the faeces of triatomine bugs, whereas *Trypanozoon*, *Duttonella*, and *Nannomonas* are salivarian, transmitted through the saliva of biting flies. Within *Trypanozoon*, *Trypanosoma equiperdum* and *Trypanosoma evansi* are considered derived forms of *T. brucei* that have undergone reductive evolution, particularly affecting mitochondrial function and the kinetoplast (kDNA), which is essential for development in the insect vector [2,3,4]. Consistent with this, *T. evansi* does not develop within an insect vector and is transmitted mechanically by biting flies, while *T. equiperdum* has lost vector transmission entirely and is now spread venereally.

Within the domain Diaphoretickes, the relevant lineages belong to the phylum Apicomplexa, mainly within the class Aconoidasida (orders Haemosporida and Piroplasmorida). To the order Haemosporida belong the genera *Plasmodium* and *Leucocytozoon*. Species of the genus *Plasmodium* are capable of infecting a wide range of vertebrate hosts, though individual species are typically restricted to a limited host range: for example, *Plasmodium falciparum*, *Plasmodium malariae*, and *Plasmodium vivax* infect humans, while *Plasmodium gallinaceum* and *Plasmodium juxtanuclare* are avian parasites; the systematics of the order as a whole has been comprehensively reviewed by Perkins [5]. *Leucocytozoon* spp. infect a broad diversity of bird taxa, although individual species are typically restricted to relatively narrow host ranges. To the order Piroplasmorida belong *Babesia* spp. and *Theileria* spp., whose individual species are also restricted to certain hosts, with many being important pathogens of livestock and companion animals. A non-aconoidasidian digenetic pathogen is *Hepatozoon* spp., which belongs to the class Conoidasida of the phylum Apicomplexa (order Eucoccidiorida). The genus has a broad vertebrate host range that includes reptiles, amphibians, and rodents; in the veterinary setting, however, the species of clinical relevance—such as *Hepatozoon canis*, *Hepatozoon americanum*, and *Hepatozoon felis*—primarily affect the order Carnivora, including dogs, cats, and wild carnivores [6]. *Hepatozoon* is acquired by the vertebrate host through ingestion of an infected tick or other arthropod—most notably *Hepatozoon canis* in dogs—a transmission route that applies broadly across the genus and constitutes an important diagnostic and epidemiological consideration [6].

The geographical distribution of the infections caused by these organisms is not arbitrary, being limited by the distribution of arthropod vectors and the availability of competent vertebrate hosts. Some parasites circulate in complex ecological networks involving numerous reservoir hosts and vector species. For example, *T. cruzi*, the causative agent of Chagas disease, infects more than 100 mammalian species and is transmitted by multiple triatomine bug genera, allowing the parasite to persist in sylvatic, peridomestic, and domestic cycles across the Americas [7]. Similarly, *Leishmania infantum* is transmitted by several phlebotomine sand fly species and maintained primarily in dogs but also in wildlife reservoirs [8,9]. In contrast, other protozoa exhibit more constrained transmission systems. Piroplasms such as *Babesia bovis* and *Babesia canis* depend on specific tick vectors and relatively limited host ranges [10,11], while *H. canis* and *Hepatozoon americanum* require ingestion of infected ticks and are therefore closely linked to particular host–vector ecological interactions [6]. These differences in host and vector diversity determine that, for a correct diagnosis, it is essential to evaluate the species of the animal affected and the specific geographic location where the animal lives or has come from (Supplementary Table S1). For example, *B. bovis* and *Babesia bigemina* primarily affect cattle, *Theileria equi* affects equids, while *L. infantum* principally parasitises dogs (though it can also infect cats, humans, and various wildlife species), and *Cytauxzoon felis* is restricted to felids [12,13]. In a similar fashion, a trypomastigote detected in a cow in sub-Saharan Africa is most likely an African animal trypanosome—most commonly *Trypanosoma congolense* or *Trypanosoma vivax*, but potentially *Trypanosoma brucei*—with species-level discrimination requiring molecular confirmation.

Within the following sections, the principal microscopic features used for detection and identification of these parasites are discussed, together with the serological and molecular tests that can confirm and refine the diagnosis. Finally, the importance of accurate vector-borne disease diagnosis within the framework of One Health is discussed.

## 2. Microscopic Diagnosis

Microscopic examination is the first-line approach for diagnosing vector-borne protozoal infections and can provide accurate identification, especially at the genus level, although determining the specific species can require additional tests. Among its advantages are low cost, rapid turnaround, and direct visualisation of the parasite within host cells, providing both diagnostic confirmation and morphological information unavailable from indirect methods. However, accuracy is highly dependent on the examiner’s expertise, particularly their knowledge of parasite morphology across different developmental stages and tissue distributions. Diagnosis is especially challenging when the parasitic load is low and can be easily missed if parasitic disease is not high on the differential diagnosis or if too few microscopic fields are examined. When the diagnosis is suspected but there are few parasites, concentration assays such as quantitative buffy coat (QBC) should be employed [14]. QBC is particularly useful for leukocyte-associated parasites (e.g., *Hepatozoon* spp.) and extracellular trypanosomes; it is not suited for intraerythrocytic organisms such as *Babesia* spp., *Theileria* spp., or *Cytauxzoon* spp., for which thick and thin blood smears remain the method of choice.

**Figure 2:**
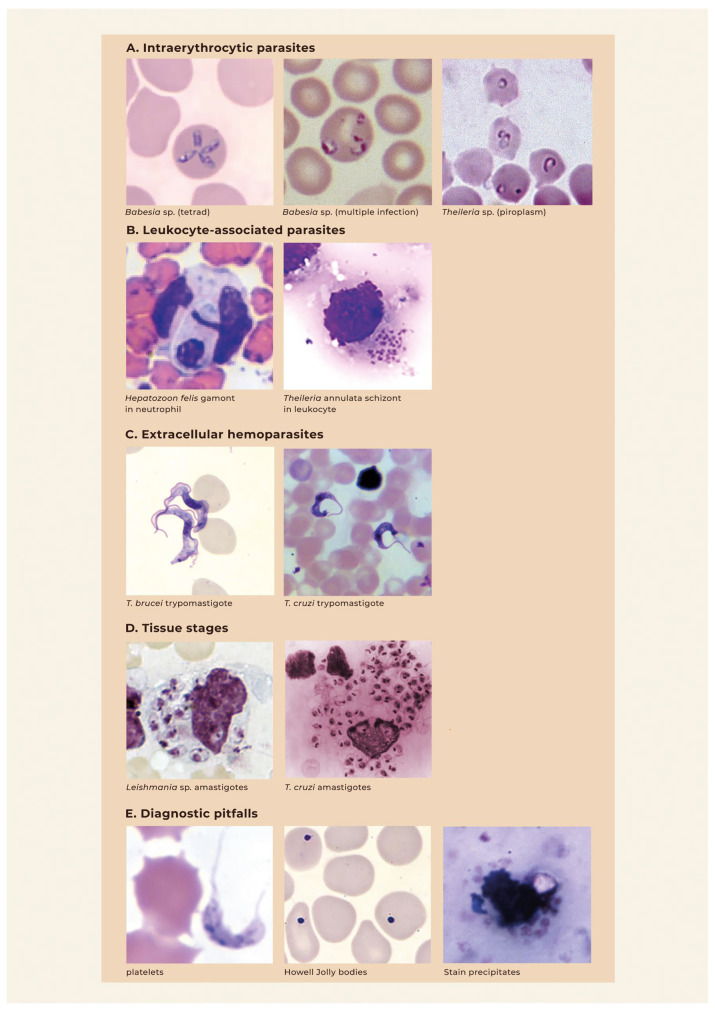
Comparative microscopic features and diagnostic pitfalls of vector-borne protozoa relevant to veterinary diagnosis. (**A**) Intraerythrocytic parasites illustrating morphological overlap among piroplasms, including *Babesia* spp. (tetrad “Maltese cross” formation and multiple infections of erythrocytes) and *Theileria* spp. (piroplasms within erythrocytes); (**B**) Leukocyte-associated parasites demonstrating host cell tropism. *Hepatozoon* spp. gamonts (represented here by *Hepatozoon felis*) are observed within neutrophils, whereas *Theileria annulata* schizonts are present within leukocytes. (**C**) Extracellular hemoparasites showing the trypomastigote morphology of *Trypanosoma* spp., highlighting differences between *Trypanosoma brucei* (elongated form with a well-developed undulating membrane) and *Trypanosoma cruzi* (prominent kinetoplast and typically curved body); (**D**) Tissue-stage parasites showing intracellular amastigotes of *Leishmania* spp. within macrophages and *Trypanosoma cruzi* within a bladder cell; (**E**) Diagnostic artefacts in blood smears that may be misinterpreted as protozoal parasites. Examples include a platelet mimicking an extracellular parasite, erythrocytes containing Howell–Jolly bodies resembling intraerythrocytic organisms, and stain precipitate forming irregular, non-cellular deposits. Images in panels (**A**,**C**–**E**) are courtesy of the Centers for Disease Control and Prevention (CDC), Public Health Image Library (PHIL), and DPDx and are in the public domain. The *Hepatozoon felis* image in panel (**B**) is reproduced from Baneth et al. (2013) [15], distributed under the Creative Commons Attribution (CC BY 2.0) licence. The *T. annulata* schizont image in panel (**B**) is reproduced from Branco et al. (2010) [16], with permission of the authors, under the Creative Commons Attribution Non-Commercial (CC BY-NC 4.0) licence.

The choice of specimen is guided by the biology of the suspected parasite. Blood samples are used to identify *Plasmodium* spp., *Babesia* spp., *Theileria* spp., *Cytauxzoon* spp., *Hepatozoon* spp. and *Trypanosoma* spp. For infections in which the parasite preferentially resides in tissues rather than blood, such as *Leishmania* spp. and *T. equiperdum*, examination of biopsy material is necessary (Figure 2).

### 2.1. Blood Smear Examination

Thick and thin blood smears are the primary diagnostic tests done when a blood parasitic infection is suspected. In a recent study conducted in a low-prevalence human hospital setting, it was demonstrated that routine haematology thin smears can also be used to detect parasites, suggesting that careful examination of the routine haematology smear could yield a diagnosis even when infection is not initially suspected [17]; whether this finding translates to veterinary practice—where parasite species, prevalence baselines, host blood-cell morphology, and laboratory workflows differ markedly from human medicine—warrants systematic evaluation in veterinary-relevant settings before routine adoption can be recommended. Among the pathogens detectable in blood, *Plasmodium* spp., *Babesia* spp., *Theileria* spp., and *Cytauxzoon* spp. have intraerythrocytic stages. *Theileria* spp. and *Cytauxzoon* spp. also have stages that affect white blood cells, whereas *Hepatozoon* spp. are intra-leukocytic (gamonts within neutrophils and monocytes). In contrast, trypanosomes are extracellular. Correct identification requires familiarity with the morphological features of each genus at the stages present in peripheral blood. In this section some of the main characteristics that give information about the identification of specific species are detailed, with data centred on species that affect veterinary companion and livestock animals rather than humans.

The erythrocytic stages of avian *Plasmodium* species show marked variation in developmental patterns across hosts, requiring an experienced observer for correct identification. A key diagnostic feature is the presence of sexually dimorphic gametocytes (macro- and microgametocytes), which are readily identifiable under the light microscope and clearly distinguish avian malarial parasites from other intracellular apicomplexans, such as *Babesia* spp., *Theileria* spp., and *Cytauxzoon* spp. [18]. An additional finding is the accumulation of hemozoin—residual pigment granules resulting from incomplete haemoglobin digestion—which is birefringent under polarised light and serves as a reliable feature distinguishing haemosporidian parasites from piroplasms, which lack hemozoin.

In contrast to *Plasmodium* spp., *Babesia* spp. are obligate intraerythrocytic parasites with very limited extra-erythrocytic development, confining their diagnostic stages almost entirely to red blood cells. Based on their morphology, *Babesia* spp. are classified as small babesias (1.0–2.5 μm long), such as *B. bovis*, *Babesia gibsoni*, *Babesia silvestris* and *Babesia rodhaini*, and large babesias (2.5–5.0 μm long), such as *B. bigemina*, *Babesia caballi* and *B. canis*. Although pleomorphic, characteristic morphological features include paired pyriform (pear-shaped) merozoites and, in some species, the tetrad form (“Maltese cross”). Among veterinary piroplasms, this form is most characteristic of *T. equi* (formerly *Babesia equi*); it is also well described in *Babesia microti*, a rodent parasite and zoonotic agent, where it represents a key diagnostic feature. Unlike *Plasmodium* spp., *Babesia* spp. lack hemozoin and do not form gametocytes in the vertebrate host, which helps distinguish them in blood smear examination [19]. Although morphological features remain the foundation of microscopic *Babesia* identification, overlapping appearances between closely related species—and between *Babesia* and *Theileria* piroplasms—can limit species-level resolution on blood smears, and molecular confirmation is recommended where species discrimination is clinically or epidemiologically important.

In peripheral blood smears, the diagnostic stages of *Theileria* spp. are intraerythrocytic piroplasms that appear as small rings which form morphologically very similarly to those of *Babesia* spp. Unlike *Babesia* spp., however, *Theileria* spp. additionally undergo a schizogonous stage within leukocytes, and this leukocyte-stage schizont—when identifiable in lymph node aspirates or impression preparations—is a useful differential feature. Different species exhibit characteristic schizont host–cell tropisms: *Theileria parva* preferentially infects T lymphocytes (including CD4+, CD8+, and γδ T-cell subsets), whereas *T. annulata* primarily infects B cells and monocytes/macrophages [20,21,22,23]; *T. equi* has a broader tropism, infecting B lymphocytes, T lymphocytes, and monocytes/macrophages in vitro, although experimental evidence indicates that monocytes/macrophages are the functionally critical host cell in vivo [24].

Closely related to *Theileria*, *Cytauxzoon* belongs to the family Theileriidae, and their piroplasm stages in the erythrocytes of mammalian hosts are morphologically very similar. The two differ, however, in the location of schizogony: in *Cytauxzoon* spp., it occurs in macrophages, whereas in *Theileria* spp. it takes place in lymphocytes (e.g., *T. parva*) or macrophages (e.g., *T. annulata*). *Cytauxzoon* has a schizogonous tissue phase in addition to an intraerythrocytic piroplasm phase, characterised by massive proliferation of schizonts within macrophages lining the vascular endothelium of multiple organs. When tissue involvement is suspected, aspiration cytology or impression smears of affected organs and histological sections are required [13].

Unlike the apicomplexans discussed above, *Hepatozoon* is not intraerythrocytic at any blood stage. The diagnostic stage in peripheral blood is the gamont—a large, elongated organism enveloped in thick membranes found within the cytoplasm of neutrophils and occasionally monocytes—which is morphologically distinct from the intraerythrocytic ring forms of *Babesia* spp., *Theileria* spp., and *Cytauxzoon* spp. and, therefore, unlikely to cause confusion in a well-prepared blood smear. Gamonts of *H. americanum*, *H. felis* and *Hepatozoon silvestris* can also be identified, but parasitemia is low and buffy coat smears are needed to increase sensitivity [25].

Turning to the extracellular trypanosomes, the detection rate varies considerably across species due to differences in vascular localisation. The species *T. congolense* sequesters to the vascular endothelium, and *T. vivax* has recently been shown to display similar behaviour in experimental mouse models [26]; whether this is a species-wide feature or a strain-specific phenomenon in veterinary-relevant hosts remains to be determined. For *T. congolense*, bioengineered microvascular models have demonstrated that the vast majority of parasites adhere to the endothelium of multiple organs rather than circulating freely [27]. When peripheral parasitemia is low, concentration methods such as the miniature anion-exchange centrifugation technique (mAECT) or the quantitative buffy coat (QBC) method substantially improve detection sensitivity for trypanosomes [28]. By contrast, *T. evansi* circulates freely and yields high peripheral parasitaemia, making smear detection straightforward, though ocular and cerebrospinal fluid localization in late disease means blood smears do not reflect total parasite distribution. The species *T. brucei* circulates in blood but additionally establishes extravascular reservoirs in tissues such as skin, adipose tissue, and muscle by crossing the vascular endothelium; these reservoirs contribute to relapsing parasitaemia and may reduce smear sensitivity at low-burden time points. Among the salivarians, *T. equiperdum* presents the greatest diagnostic challenge: its primary localization in genital mucosa, skin, peripheral nerves, and the distal spinal cord means peripheral parasitemia is consistently and profoundly low. Blood smear examination was negative in all naturally infected horses despite confirmed infection by PCR and histopathology [29]. Blood smear examination therefore has no meaningful negative predictive value for *T. equiperdum* and cannot be relied upon to exclude infection. Finally, *T. cruzi*, a stercorarian trypanosome, differs from the salivarian species in that during the acute phase free-circulating parasites are detectable; in the chronic phase, however, the parasite load in blood becomes negligible, and therefore blood smears will not be diagnostic.

Across all of these parasite groups, a negative microscopy result does not exclude infection in low-parasitaemia, chronic, tissue-localised, or carrier states; clinical suspicion supported by host species, geographic origin, and clinical presentation should therefore trigger serological or molecular follow-up regardless of an unremarkable smear.

### 2.2. Tissue-Stage Parasites and Exo-Erythrocytic Development

Although the majority of these pathogens can be detected in blood, they can also present developmental stages in other tissues that require tissue examination for detection. Others, such as *Leishmania* spp., are not present in blood.

In avian malaria, erythrocytic merozoites can trigger secondary tissue merogony—a process of exo-erythrocytic replication occurring in endothelial cells, macrophages, and erythrocyte precursors, depending on the parasite lineage, and detectable in impression smears or histological sections of affected organs.

*Hepatozoon* species also exhibit diagnostically relevant tissue stages. Extravascular meronts of *H. canis* are detectable in cytological preparations from lymph nodes, spleen, and bone marrow, appearing round to oval with merozoites arranged peripherally around a central core, producing a characteristic “wheel-spoke” pattern [25]. In contrast, meronts of *H. americanum* and *H. felis* localise primarily to striated muscle (skeletal and cardiac), as does *H. silvestris*. Similarly, *C. felis* develops a macrophage-associated schizogonous stage in organs such as the spleen, liver, lungs, and lymph nodes, where enlarged parasitized macrophages obstruct small vessels and sinusoids, causing ischaemia and severe systemic inflammation. These stages are best identified by fine-needle aspiration cytology of affected organs, which reveals schizont-laden macrophages, in impression smears or histological sections rather than in peripheral blood [30].

In *Leishmania* infections, tissue tropism reflects the clinical form of disease. Cutaneous leishmaniosis involves dermal macrophages, while visceral disease disseminates to the spleen, liver, and bone marrow. In all forms, the diagnostic stage is the amastigote—a small, oval, non-flagellated organism residing within the parasitophorous vacuole of the macrophage, visible on Giemsa-stained tissue impressions or histological sections. Examination of affected tissues reveals macrophages packed with intracellular amastigotes, the so-called Leishman–Donovan bodies, which are the hallmark microscopic finding in parasitological diagnosis [31,32].

Similarly, requiring tissue examination in its chronic form, *T. cruzi* infection is characterised during the acute phase by detectable parasitemia, but in the chronic phase, the parasite localises in target organs such as the heart and intestines. A biopsy of cardiac or skeletal muscle in chronic Chagas disease may reveal intracellular amastigote nests, though tissue sampling is invasive and rarely performed in routine diagnosis.

### 2.3. Advances in Artificial Intelligence for Diagnosis of Parasitoses in Clinical Samples

With the development of strong algorithms for the analysis of medical images to improve diagnosis, efforts are being made to develop algorithms for the detection and classification of parasites, with the majority of studies being done for the diagnosis of human malaria infection [33,34]. In veterinary practice, there is a commercially available platform for the detection of intestinal parasites in the faeces of cats and dogs that performs with a sensitivity and specificity of greater than 90%, with the exception of the diagnosis of *Ancylostoma* in cats, which is approximately 80% [35]. For the diagnosis of vector-borne parasites, there are only a few studies that have shown the potential of artificial intelligence (AI)-assisted microscopy for use in veterinary practice, with algorithms for trypanosomes less developed and based either on experimental infection or on mixed data from humans and animals (Table 1).

However, the accuracy values reported in Table 1 reflect performance on curated research datasets and have not yet been validated in routine clinical or field practice. Current datasets are small, typically built from single-erythrocyte images rather than whole-smear samples, and the impact of variability in staining protocols, slide preparation, and microscope optics on accuracy has not been systematically evaluated. Existing tools are also assistive rather than autonomous—an initial clinical suspicion is still required to trigger their use—and ideally, they would be integrated into routine smear analysis rather than reserved for suspected cases. Image acquisition in low-resource settings often depends on smartphone adapters, and coordinated efforts to expand curated public repositories would help address data scarcity. Making validated algorithms freely available as downloadable software or apps would further accelerate uptake [43].

## 3. Serological Diagnosis of Vector-Borne Protozoal Infections

### 3.1. The Role of Serology in Vector-Borne Protozoal Diagnosis

Serology occupies a central place in the diagnosis and surveillance of vector-borne protozoal infections of veterinary importance. The reasons are practical: parasitaemia in many of these infections is low, intermittent, or below the detection limit of direct microscopy—the chronic phase of *Trypanosoma cruzi* (very low and intermittent peripheral parasitaemia), *T. equiperdum* (parasites confined to genital and neural tissues), *Leishmania* spp. infections (amastigotes generally absent or only rarely detectable in peripheral blood), and the carrier state in bovine babesiosis and theileriosis (low parasitaemia maintained by partial immune control of replication)—making direct microscopic detection insensitive. Serological assays detect host antibodies raised against parasite antigens and remain informative in precisely those situations where the parasite itself is not readily found [44]. They are easy to use, scalable, and capable of detecting prior exposure in asymptomatic or subclinical animals [45], and combined formats are increasingly available for the simultaneous detection of multiple co-circulating pathogens, reflecting the frequent occurrence of co-infections [46,47]. Serology is also widely available across the four parasite groups reviewed here, with mature reference standards: World Organisation for Animal Health (WOAH)-prescribed competitive ELISA (cELISA) for international trade certification of equine piroplasmosis [48]; WOAH manual standards for bovine babesiosis and theileriosis; and the LeishVet guidelines for clinical staging of canine leishmaniosis [49]. These assays exist in both laboratory-based and point-of-care (POC) formats, which serve genuinely different use-cases: clinical confirmation in the individual animal, herd-level screening, and population-level epidemiological surveillance. The remainder of this section is organised around these three roles, followed by a structured account of the principal limitations of serological testing and how to interpret a serological result in light of them.

A practical implication of the quantitative outputs of laboratory-based assays—titres or optical density values—is that a paired or serial sera can, under certain conditions, address a limitation that no single time point can: the inability to distinguish active recent infection from previous exposure. The diagnostic value of titre dynamics has been most clearly demonstrated for canine leishmaniosis, where longitudinal IFAT and ELISA studies show that rising titres track progression and clinical relapse, while falling titres accompany successful treatment response [50,51]; fluctuating titres in subclinically infected dogs reflect the complex host–parasite dynamics characteristic of chronic carriage. In canine *T. cruzi* infection, longitudinal cohort studies document seroconversion as a sensitive marker of new infection [52], although once the carrier state is established, antibody titres remain stable for years to decades, and the active-versus-past distinction by serology is not informative on practically useful timescales [53]. For bovine babesiosis and theileriosis, persistent carrier states with antigenic variation mean that antibodies are detectable for many months to years after acute infection and titre dynamics are correspondingly uninformative [54]. For African and South American animal trypanosomiases, longitudinal antibody-titre data in natural infections are notably scarce, and the diagnostic value of paired sera in this group remains to be established. Where validated molecular assays are unavailable, serial or paired-serum serological testing may provide a practical complementary approach for the diagnosis of canine leishmaniosis and acute *Trypanosoma cruzi* infection, although these strategies remain insufficiently standardised and warrant further systematic evaluation in veterinary practice.

### 3.2. Serology in Clinical Practice

At the level of the individual animal, serology is most useful for confirming infection in clinical contexts where direct parasite detection is unreliable. This includes chronic *T. cruzi* infection (the indeterminate and determinate phases, where parasitaemia is low and intermittent), *T. equiperdum* infection (where parasites are confined to genital and neural tissues), and *Leishmania* spp. infections (where amastigotes are generally absent or only rarely detectable in peripheral blood). For these and for any infection in which parasitaemia is low or intermittent, serological testing supports clinical suspicion and contributes to diagnosis when interpreted alongside host species, clinical signs, and geographic origin (Supplementary Table S1).

Two formats serve clinical practice. Laboratory-based assays—principally ELISA and IFAT—provide quantitative or semi-quantitative outputs (titres, optical density values) essential for clinical staging and longitudinal monitoring [55]. The IFAT method is commonly regarded as a reference for validation of new diagnostic tools and is the reference method for canine leishmaniosis [55,56]. ELISA is preferred over IFAT for bovine theileriosis and babesiosis when throughput matters, principally because the platform is more amenable to automation and large-scale screening [57]. Point-of-care (POC) tests—lateral flow assays and rapid diagnostic tests—detect antibodies, or occasionally antigens, using colloidal gold visible to the naked eye within 10–20 min without the need for laboratory equipment or trained personnel [58]. They are useful for sampling animals in remote locations and as rapid tools in veterinary clinics [59], can be performed on-site or later by elution of dried blood spots from filter paper [60], and are available in formats that detect antibodies against multiple co-circulating pathogens simultaneously—addressing the frequent occurrence of co-infections in vector-borne disease.

The rK39-based lateral flow test for canine visceral leishmaniosis shows high sensitivity and specificity approaching 100% in some validation studies [55,61], although performance is not uniform across endemic settings: in Colombia, two immunochromatographic tests showed sensitivity of 82.9–85.7% and specificity of 79.6–92.6% [62], with both missing *Leishmania braziliensis* and *L. amazonensis* cases—illustrating the risk of poor performance when local species differ from the test antigen’s target. In equids, combined POC tests permit simultaneous serological detection of antibodies to *T. equi* and *B. caballi*, a relevant capability given that the two agents differ in drug susceptibility [63]; however, reliable discrimination between concurrent infections depends on platform performance and cross-reactivity, which vary across products. The Card Agglutination Test for Trypanosomiasis (CATT/*T. evansi*) targets the RoTat 1.2 variant surface glycoprotein and shows high sensitivity in horses, camels, goats, sheep, buffaloes, and dogs but lower sensitivity in cattle and pigs. CATT performance is constrained by parasite biology because the assay detects primarily *Trypanosoma evansi* Type A strains expressing the RoTat 1.2 antigen. Type B isolates described to date lack the RoTat 1.2 gene and therefore escape detection. Similarly, certain dyskinetoplastic lineages—which have partially or completely lost their kinetoplast DNA (kDNA; discussed in Section 4)—have been associated with the absence of RoTat 1.2 expression and may yield false-negative results. Importantly, this limitation does not reflect recognition of a kDNA-encoded antigen, as RoTat 1.2 is nuclear-encoded, but rather the empirical association between certain dyskinetoplastic lineages and absence of RoTat 1.2 expression. Consequently, a negative CATT result does not exclude *T. evansi* infection in regions where Type B strains circulate or where dyskinetoplastic stocks are suspected. For bovine babesiosis there are also rapid diagnostic tests used for quick screening of the herd. Rapid tests, while convenient, often exhibit lower analytical sensitivity than laboratory-based assays, and their utility for international certification remains limited, with ELISA and IFAT being the current standards [48]. Examples of serological tests available for key vector-borne protozoal parasites, including the antigens used, platforms, and test types, are summarised in Supplementary Table S2; the list is illustrative rather than exhaustive.

### 3.3. Serology for Herd Management and Surveillance

Beyond the individual case, serology is the practical workhorse of herd-level screening and population-level surveillance. The reasons are operational: laboratory-based serology can be standardised, scaled, and applied to large numbers of samples at modest cost per sample, and quantitative outputs allow tracking of seroprevalence over time and across regions. For equine piroplasmosis, WOAH-prescribed cELISA is the regulatory standard for international trade movement of horses, and its use harmonises serological reporting across countries [48]. Surveillance of canine leishmaniosis in endemic Mediterranean and Latin American regions has historically relied on serological methods, particularly ELISA and IFAT, frequently based on whole-promastigote or soluble promastigote antigens, to estimate seroprevalence and monitor changes associated with control interventions [64].

An important but still underdeveloped contribution of herd-level and surveillance serology is the cross-geographical evaluation of diagnostic assay performance. Applying validated serological assays across multiple endemic settings through coordinated surveillance networks would allow assessment of how test sensitivity and specificity vary under differing transmission intensities, host assemblages, and circulating parasite populations. Such approaches would provide more realistic estimates of field performance than single-region validation studies, whose findings may not generalise across epidemiological contexts, as demonstrated for several protozoal serological assays [65,66,67]. Coordinated evaluation of this type aligns naturally with the One Health surveillance frameworks discussed in Section 5.

The need for cross-geographical assay evaluation is particularly acute for parasites with substantial genetic and antigenic diversity. The species *T. cruzi* is organised into seven Discrete Typing Units (DTUs: TcI–TcVI and TcBat), and the sensitivity of serological diagnostic tests varies considerably across DTUs, while specificity is frequently affected by cross-reactivity with other trypanosomes or with *Leishmania* spp. TcI is the most widely distributed in wildlife and domestic animals across Latin America; TcII, TcV, and TcVI predominate in domestic transmission cycles in the Southern Cone; and TcIV is associated with wildlife reservoirs in both North and South America. DTU typification is not possible through serological methods, and Chagas disease serodiagnosis is therefore conventionally based on a combination of at least two different tests; however, the choice of which tests to combine is not globally standardised and depends on the platforms validated for each region [68]. This is a worked example of why surveillance-grade assay performance for any one parasite group cannot be assumed to generalise across the parasite’s geographic range.

### 3.4. Interpreting a Serological Result: Four Limitations

The interpretation of any serological result is constrained by limitations that vary in importance by parasite, host, and epidemiological setting. Four limitations are particularly consequential for vector-borne protozoal infections.

Persistent antibodies after treatment, apparent parasite suppression, or carrier states. In several of the parasites reviewed here, antibodies remain detectable long after the acute phase has resolved or peripheral parasitaemia has been controlled. For *Trypanosoma cruzi*, infection is essentially lifelong in the absence of effective treatment, antibodies persist accordingly, and even after antitrypanosomal treatment, the time to negative seroconversion—which is itself an imperfect surrogate for parasitological cure—is measured in years to decades and is inversely proportional to the pretreatment duration of infection [53]. For bovine babesiosis, recovered cattle remain symptomless carriers for months to years (longer for *B. bovis* than for *B. bigemina*), and antibody titres are not a reliable indicator of current infection status: cattle that have become serologically negative on indirect haemagglutination testing have been shown to remain immune four years after initial vaccination or exposure, while cattle that have eliminated the parasite may continue to test positive [54]. Carrier states in bovine babesiosis and theileriosis are characterised by low parasitaemia resulting from partial immune control of parasite replication; the balance between parasite persistence mechanisms—including antigenic variation—and host splenic and adaptive immune responses permits long-term subclinical infections that serve as epidemiologically important reservoirs [69]. The general principle is that the presence or absence of detectable antibodies at a single time point reflects past immunological exposure, not necessarily ongoing parasite replication or current parasitaemia.

High background seroprevalence in endemic settings. Where transmission has been sustained for years, a substantial fraction of apparently healthy animals carry antibodies to the locally circulating parasites. This is the operational reality of endemic stability for bovine babesiosis and theileriosis: classically associated with high early-life exposure rates (often >70–75% of calves exposed to *B. bovis* before nine months of age) and a low incidence of clinical disease in older cattle, the dynamic equilibrium produces high herd-level seroprevalence in clinically unaffected populations [54]. The same dynamic occurs in canine leishmaniosis in the Mediterranean basin and in Brazilian foci, where seroprevalence in apparently healthy dogs can be substantial. In such settings, a positive serological result on a single sample carries limited specificity for clinically relevant infection, and interpretation must explicitly account for the local prevalence baseline. In settings where *Leishmania* spp. and *Trypanosoma cruzi* co-circulate, limited but measurable serological overlap has been documented: in rural Panamanian dogs, agreement between *Leishmania panamensis* IFAT and *T. cruzi* IFAT or multiplex microsphere immunoassay was low but detectable (κ = 0.18–0.20), illustrating the diagnostic complexity of interpreting serology in regions with sympatric trypanosomatid transmission [67].

Vaccine-induced seroconversion. Where vaccines have been deployed, vaccine-induced antibodies can interfere with serological diagnosis in ways that depend critically on the assay platform and the vaccine in use. For canine leishmaniosis, CaniLeish^®^ induces antibodies that cross-react with whole-promastigote–based assays such as IFAT, DAT, and crude soluble-antigen ELISA: in a controlled longitudinal study, 74.1% of CaniLeish-vaccinated dogs were classified as seropositive by a whole-promastigote ELISA one month after completion of the primary vaccination course [70]. Recombinant rK39- and rK28-based ELISAs are less affected, with vaccinated non-infected dogs in a 25-month longitudinal cohort showing serological profiles statistically indistinguishable from non-vaccinated non-infected controls, although the cohort was small and individual false-positive results were observed [71]. Letifend^®^, a recombinant-protein vaccine designed under the Differentiating Infected from Vaccinated Animals (DIVA) principle, has shown reduced interference with several commonly used serological assays in vaccinated non-infected dogs, although the extent of non-interference is platform-dependent and independent peer-reviewed replication remains limited [72]. The practical consequence is that, in CaniLeish-vaccinated populations, whole-antigen serology cannot reliably distinguish vaccinated from infected animals, and confirmation of infection generally requires molecular detection of parasite DNA. A parallel issue arises for cattle babesiosis and theileriosis, where live, attenuated, and soluble-antigen vaccines have been in use for decades: vaccinated cattle seroconvert against the same antigens used in diagnostic assays, and serological assays cannot distinguish vaccinated from naturally infected animals in a single sample [73]. The DIVA principle has therefore become an explicit design objective for next-generation veterinary protozoal vaccines, and vaccination history must be a routine part of the clinical and epidemiological information accompanying any serological request in regions where these vaccines are deployed.

Antibody titre does not reliably correlate with parasitaemia or infectivity. Even where serology unambiguously confirms infection, the antibody titre itself is an unreliable proxy for parasite burden, ongoing transmission risk, or infectivity to the vector. Outside specific contexts such as the clinical staging of canine leishmaniosis—where titre magnitude does correlate moderately with disease severity, though not necessarily with infectivity—titres should not be interpreted as a quantitative readout of parasitaemia. This limitation underlies the need for adjunct molecular or parasitological testing whenever questions of active parasite replication, treatment response, or transmissibility are clinically or epidemiologically relevant—and it is the reason that surveillance programmes increasingly combine serological screening with molecular confirmation rather than relying on serology alone.

Together, these four limitations mean that a serological result is best interpreted not in isolation but within a structured framework that integrates host species and breed, geographic origin, clinical presentation, vaccination history, regional seroprevalence baseline, and, where available, paired-sera dynamics or molecular confirmation.

### 3.5. Implications for Surveillance in Vaccinated Populations

The growing use of canine leishmaniosis vaccines in endemic regions raises a structural question for surveillance. Where whole-antigen ELISA or IFAT has historically been the workhorse of population-level seroprevalence monitoring—across the Mediterranean basin and in Latin American foci—increasing CaniLeish^®^ coverage will introduce a growing fraction of vaccinated animals whose serology is indistinguishable from naturally infected animals on these platforms [70]. The implication is that traditional seroprevalence-based surveillance becomes progressively less informative about *Leishmania* infection dynamics as vaccination coverage rises and that molecular surveillance, recombinant-antigen-based serology with documented absence of vaccine cross-reactivity, or DIVA-compatible vaccines (such as Letifend^®^, where independent validation continues to accrue) become correspondingly more important. A parallel issue arises for any vector-borne protozoal infection where vaccination is or becomes available—including bovine babesiosis and theileriosis, where the lack of DIVA-compatible vaccines limits the diagnostic resolution of post-vaccination surveillance: the reliability of seroprevalence as a surveillance indicator depends on the assay platform, the vaccine antigen, and the regional vaccination coverage.

A parallel surveillance gap exists for *T. cruzi* in veterinary settings. Commercially available serological tests were originally developed for human use and are widely implemented in hospital blood transfusion and organ transplant screening programmes in endemic regions [74]. The limited application of serodiagnosis in livestock is partly explained by the prolonged indeterminate phase of Chagas disease, characterised by low parasitaemia and the absence of clinical signs: production animals usually have relatively short productive lifespans and are unlikely to develop chronic disease. Despite this, the recognised role of dogs as important reservoirs and sentinels in the domestic transmission cycle of *T. cruzi* [75] underscores the need for diagnostic tools and integrated screening programmes that include both humans and companion animals within a One Health framework. These considerations are taken up further in Section 5.

## 4. Molecular Diagnosis

### 4.1. Target Selection

The detection of the DNA of the pathogen is ideal, as it demonstrates the presence of an active infection rather than the demonstration of antibodies, which indicate exposure to the parasite. The selection of an assay for the molecular identification of any infectious agent is based on the choice of a specific target to detect, the availability and cost of the method needed to detect it, and the reasons behind testing (individual case diagnosis vs. surveillance). Target selection is a balance between the sensitivity, specificity and epidemiological utility of the test. The most widely used targets are highly conserved sequences present in multiple copies in the pathogen genome, such as ribosomal RNA (rRNA) genes, mitochondrial DNA and repetitive elements. The greater the number of copies of a given target, the greater the sensitivity of the assay. For example, differences in copy number determine that in *C. felis*, PCR amplification of the cytochrome c oxidase subunit III gene (*cox3*) is more sensitive than amplification of the 18S gene [76].

**Figure 3:**
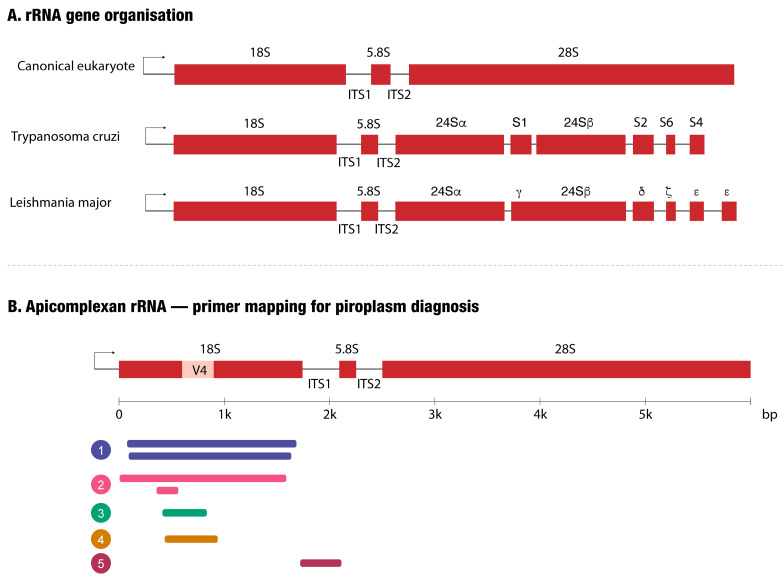
Ribosomal RNA gene organisation in trypanosomatid and apicomplexan vector-borne protozoa and primer mapping for piroplasm molecular diagnosis. (**A**) Schematic comparison of the rRNA transcription unit. In the canonical eukaryotic cassette, the 18S, 5.8S and 28S rRNA genes are co-transcribed and separated by the internal transcribed spacers ITS1 and ITS2. In trypanosomatids the large-subunit equivalent is fragmented post-transcriptionally into multiple small RNA species: 24Sα, S1, 24Sβ, S2, S6 and S4 in *Trypanosoma cruzi* and 24Sα, γ, 24Sβ, δ, ζ and two ε species in *Leishmania major* [77]; (**B**) Primer pairs from five published assays mapped onto the apicomplexan rRNA locus, drawn to scale from the *Theileria parva* Muguga 18S reference sequence (1744 bp; gene fragments to scale across the full ~6 kb cassette). The V4 hypervariable region of 18S is highlighted in amber. Numbered, colour-coded bars indicate amplicon position and size; bars stacked in the same colour represent the two rounds of a nested PCR (outer bar above, nested inner bar below). (1) A near-full-length 18S nested PCR [87]. (2) A full-length 18S outer round, followed by a short, nested fragment upstream of V4 [88]. (3) A single-round 18S amplifying V4 [89]. (4) A single-round 18S V4 PCR coupled to targeted amplicon deep sequencing [90]. (5) A multiplex PCR for detection of four *Babesia* spp. targeting the ITS1 region [86]. Detailed primer sequences, per-species amplicon sizes and additional references are provided in Supplementary Table S3.

The rRNA genes encode ribosomal RNAs, which constitute the structural and catalytic core of the ribosome. They are usually present in tandem clusters that encode the 18S, 5.8S, and 28S rRNA subunits, whereas the 5S rRNA is encoded at a separate locus. In trypanosomatids, it is the large ribosomal subunit (28S-type) that is atypical: it is encoded by multiple discrete fragments (such as 24Sα, 24Sβ, and several small subunit RNAs in *T. brucei*) that assemble into the mature large subunit, whereas the 18S subunit is structurally equivalent to that of other eukaryotes (Figure 3) [77]. The rRNA gene copy number varies widely across genera—from only 2–4 units in *Theileria* and *Babesia* spp. to over 100 in *T. cruzi*—directly influencing assay sensitivity [77]. The coding regions of all rRNA genes are highly conserved; their sequences are therefore frequently used to evaluate evolutionary relationships between organisms. The most conserved regions serve as annealing sites for universal primers, while the more variable flanking regions are exploited for the design of taxon-specific primers [78,79,80,81,82]. The internal transcribed spacer (ITS) regions evolve approximately 100 times more rapidly and are consequently more variable [83]. A combination of primers (18S-based and ITS-based) have been exploited both for the design of primers enabling simultaneous detection of several species of piroplasms, plasmodia or trypanosomes and for species identification using the more variable ITS1 and ITS2 regions. A critical limitation of 18S rRNA-based assays in Piroplasmida, however, is that diagnostic utility depends strongly on the length of the amplified fragment and the variable regions it spans. The most widely used pan-piroplasmid primers generate amplicons of approximately 320–450 bp, spanning only one or two variable regions (primarily V4) of the 18S gene (Figure 3B). Sequence divergence within V4 among closely related piroplasm species is insufficient for reliable species- or genotype-level discrimination, and similarity-based identification methods such as BLASTn may return ambiguous or misleading results when short fragments are queried.

The need for greater discriminatory power than 18S provides is exemplified by the *Theileria orientalis* complex: identification of the pathogenic Ikeda and Chitose genotypes, responsible for substantial outbreaks of bovine anaemia in Australia and New Zealand since 2006, was achieved through sequencing of the major piroplasm surface protein (MPSP) gene rather than 18S, and whole-genome analysis subsequently revealed sufficient divergence among Ikeda, Chitose, and Buffeli genotypes to warrant their reconsideration as separate species [84,85]. For applications requiring species- or genotype-level resolution in Piroplasmida, amplicons of at least 800–1000 bp incorporating the V4, V7, and V8 hypervariable regions have been used. For the detection of *Babesia* species, a multiplex PCR system targeting the ITS1 region of *Babesia divergens*, *Babesia duncani*, *B. microti* and *Babesia odocoilei* that demonstrated no cross-amplification among the *Babesia* species tested (Figure 3B, Supplementary Table S3) [86].

Other multicopy targets are sequences within mitochondrial DNA. Some organisms, such as *Plasmodium* spp. and trypanosomatids, only have one mitochondrion [91,92]. However, each of these mitochondria contains multiple copies of mitochondrial DNA, making the encoded sequences multicopy. In trypanosomatids, the mitochondrial genome is organised as kinetoplast DNA (kDNA), which contains two classes of circular molecules with strikingly different copy numbers that have direct consequences for assay sensitivity [93,94]. Maxicircles are present in only 20–50 copies per cell and encode mitochondrial proteins, including cytochrome-related genes; their low copy number limits the sensitivity of maxicircle-based assays, but the specificity of maxicircle sequences makes them useful for species discrimination—for example, the maxicircle-encoded *nad5* (NADH dehydrogenase subunit 5) gene distinguishes *T. brucei*/*T. equiperdum* from *T. evansi* [95]. Minicircles, by contrast, are present in thousands of copies per cell (typically 5000–10,000 in *Leishmania* spp.) and encode guide RNAs; this hundred-fold or greater copy number advantage over maxicircles is the direct basis for the superior sensitivity of minicircle-based assays. Conserved minicircle sequence regions are therefore the primary high-sensitivity molecular target for *Leishmania* spp. detection [96,97,98] and have also been applied to the diagnosis of *Trypanosoma caninum* [99]. In Apicomplexa, one of the more commonly targeted mitochondrial genes is cytochrome *b* [100,101,102,103,104]. Dyskinetoplastic *T. evansi* stocks, which lack kDNA entirely, cannot be detected by any kDNA-based assay and require nuclear targets.

For African trypanosomes of the brucei and congolense groups, as well as for *T. cruzi*, the equivalent high-copy diagnostic targets are nuclear repetitive sequences. In *T. brucei* spp. a 177 bp nuclear repeat and in *T. congolense*, a 369 bp nuclear repeat have been used for sensitive PCR-based detection [105], while in *T. cruzi*, a 195 bp satellite DNA repeat element present in approximately 10^5^ copies per genome—representing approximately 10% of total DNA [106]—is the most frequently used target. In trypanosomatids all mRNAs are modified by trans-splicing; the gene encoding the spliced leader sequence has multiple copies and has also been used as a diagnostic target [107]. For species-level identification, parasite-specific proteins have been used as targets, such as the variant surface glycoprotein of the RoTat 1.2 of *Trypanosoma evansi* Type A [108,109,110,111], the Tams1 gene of *T. annulata* [112] and the Rhoptry protein gene of *B. canis* [113].

### 4.2. Amplification Methodologies

With regard to methodology, conventional PCR needs a basic laboratory setting, and results can be visualised with agarose gels. Certain protocols include restriction enzyme digestion of the amplified products [114,115], and multiplex PCR can detect one or more species in a single amplification [116]. With post-run high resolution melting analysis, specific species can be determined [80,82]. Quantitative PCR (qPCR) uses either an intercalating dye such as SYBR Green or hydrolysis probes (e.g., TaqMan) [117,118,119,120]. Droplet digital PCR (ddPCR) enables absolute quantification without a standard curve, improving sensitivity for low-parasitaemia samples, and has been applied to the detection of *T. cruzi*, *C. felis*, *B. microti* and *B. duncani* [121,122,123] and for simultaneous detection of protist and bacterial infections through multiplex ddPCR [124].

Beyond PCR-based methods, targeted amplicon deep sequencing has been applied to bovine piroplasms [90] and to characterise genetic diversity within *T. orientalis* populations [125]. The nested universal parasite diagnostic assay (nUPDx) uses nested amplification of the conserved 18S ribosomal DNA (rDNA) combined with restriction enzyme digestion of host-derived sequences prior to Illumina deep sequencing, achieving a limit of detection comparable to real-time PCR while enabling simultaneous detection of multiple blood-borne parasites, including *Plasmodium* spp., *Babesia* spp. and *Trypanosoma* spp. in a single reaction [126].

Isothermal amplification protocols have been designed to be applied at the point of care. The most common assay is loop-mediated isothermal amplification (LAMP) where detection can be achieved through SYBR Green or calcein fluorescence, turbidimetry, or specific probes such as lateral flow assay strips. Recombinase polymerase amplification (RPA) employs a protein complex including a recombinase, a single-strand binding protein and a strand-displacing DNA polymerase and is typically faster than LAMP and more tolerant of inhibitors [127]. This technology has also been coupled with CRISPR–Cas12a (extension of recombinase-aided amplification with CRISPR–Cas12a detection, ERA-Cas12a), combining high sensitivity with equipment-independent readout options [128].

### 4.3. Commercially Available Assays

Although many assays have been published for the detection of vector-borne protozoa, the assays that are commercially available and standardised are considerably fewer, and routine clinical use depends heavily on regional availability. In clinical practice, samples should be sent to a reference laboratory or, where appropriate, point-of-care formats should be used when standard microscopy is not diagnostic. Commercial real-time PCR (qPCR) kits are available for *Babesia* spp., *Theileria* spp., and *Trypanosoma cruzi* from multiple manufacturers and to a lesser extent for *Cytauxzoon* spp., *Hepatozoon* spp., and the *Trypanozoon* subgenus (*T. brucei*, *T. evansi*, *T. equiperdum*), where standalone commercial kits remain comparatively scarce and laboratory-developed PCR assays still dominate routine diagnosis. Isothermal amplification formats—including LAMP and recombinase-aided amplification—are also represented and are particularly attractive for point-of-care and field-surveillance applications because they require minimal instrumentation and short turnaround times. Local availability and regulatory status (*Conformité Européenne*—In Vitro *Diagnostic*, CE-IVD vs. *Research Use Only*, RUO vs. veterinary-use-only) vary widely by region; *T. cruzi* is the only target with multiple CE-IVD-marked human IVDs. For human babesiosis and Chagas disease in the United States, diagnosis still relies predominantly on laboratory-developed tests (LDTs) at reference laboratories. Commercial molecular kits for African animal trypanosomosis exist but are predominantly RUO and not widely deployed in routine veterinary practice, and stand-alone CE-IVD or U.S. Food and Drug Administration (FDA)-cleared commercial qPCR for *T. brucei* in humans is essentially absent: HAT diagnosis still relies primarily on parasitology, CATT/Rapid Diagnostic Test (RDT) serology, and laboratory-developed PCR or LAMP performed at World Health Organization (WHO)/Foundation for Innovative New Diagnostics (FIND)-supported reference centres. A comprehensive list of commercial molecular kits is provided in Supplementary Table S2, and a reference table of published primer sets and molecular assay designs is provided in Supplementary Table S3.

## 5. Surveillance of Vector-Borne Protozoal Infections: Challenges and a One Health Perspective

One Health is a framework that recognises the interdependence of human, animal, and ecosystem health. Despite the WHO’s longstanding emphasis on preventive and primary health care, investment in prevention remains disproportionately low, accounting for roughly 3% of total health expenditure in many human health systems [129]; comparable data for veterinary systems are lacking, but the gap between surveillance investment and disease burden is similarly stark. This imbalance is particularly consequential for vector-borne and zoonotic diseases, where upstream ecological and animal health dynamics directly shape human disease risk.

At the institutional level, the Food and Agriculture Organization (FAO), WOAH, and WHO—collectively the Tripartite—have jointly addressed public health, animal health, and environmental challenges through multisectoral and transnational cooperation, most notably through the Tripartite Zoonoses Guide [130,131]. This collaborative architecture provides an important foundation, yet translating its principles into functional, integrated surveillance systems at the national and regional levels remains an ongoing challenge.

Vector-borne protozoal infections represent a particularly complex challenge at the human–animal–ecosystem interface. Surveillance data for these pathogens are accessible through several platforms: WOAH’s World Animal Health Information System (WAHIS) provides official country-level disease data; FAO’s Emergency Prevention System for Animal Health Global Information System (EMPRES-i) integrates field reports and risk mapping [132]; the Programme Against African Animal Trypanosomosis (PAAT) Atlas covers the distribution of African trypanosomiasis; and the Pan American Health Organization (PAHO) contributes regional data on zoonotic trypanosomes in the Americas. The Eukaryotic Pathogen, Vector and Host Informatics Resource Database (VEuPathDB)’s VEuMAP platform integrates epidemiological and population-level data relevant to these pathogens [133]. Despite the breadth of these resources, veterinary surveillance remains considerably more fragmented than its human health counterpart, limiting the ability to detect emerging signals before they reach human populations.

Anthropogenic changes—including land-use modification, deforestation, agricultural intensification, and urbanisation—are expanding vector habitats and reshaping the transmission cycles of pathogens such as *Theileria* spp., *Babesia* spp., trypanosomes, and *Leishmania* spp. These dynamics intensify contact between wildlife, livestock, vectors, and humans, increasing the risk of pathogen emergence and spillover. In this context, expanding knowledge of parasite diversity and host specificity in wildlife populations is central to anticipating where and how transmission will occur.

Although the presence of many of these parasites in wildlife has been documented, characterisation frequently remains at the genus level. The bat-associated trypanosomes offer a compelling illustration: bats carry at least two *T. cruzi* lineages—TcBat, which appears primarily bat-specific with only a single reported case of human infection attributed to this genotype [134], and *T. cruzi* I, which is pathogenic to humans, domestic animals, and wildlife. Reliable discrimination requires multiple molecular approaches, including small subunit (SSU) rDNA, cytochrome *b*, and histone H2B gene sequence analyses, ITS1 rDNA-based genotyping, and nuclear multilocus sequence typing (nMLST) [135,136]. This reflects a systemic limitation in wildlife surveillance, where resource constraints routinely prevent the depth of characterisation needed to distinguish epidemiologically distinct lineages.

Although humans are not natural hosts for *Babesia* spp. and *Theileria* spp., their close proximity to domestic animals has resulted in occasional human infections. This is illustrated by two recent investigations. Breitschwerdt et al. [137] documented a One Health family outbreak in which all five members of a single household and one of their dogs were infected with *Babesia divergens*-like MO-1—a zoonotic species not previously reported in dogs—with concurrent *Bartonella* and *Borrelia* co-infection in several family members; the affected individuals presented with neurological and neuropsychiatric symptoms. In a separate cohort study, Breitschwerdt et al. [138] used enrichment blood culture and droplet digital PCR to detect *Babesia* DNA, *Bartonella* DNA, or both in 23 of 50 patients presenting with chronic fatigue and concurrent neurological symptoms. Together, these studies highlight the importance of detecting mixed infections with related stealth pathogens (*Babesia*, *Bartonella*, *Borrelia*), which standard single-target diagnostic workflows readily miss.

The consequences of inadequate molecular resolution are well illustrated by studies of *Theileria orientalis*, in which standard PCR and consensus sequencing fail to resolve mixed-genotype infections. Targeted amplicon deep sequencing has revealed substantial within-host and within-herd genotype diversity, indicating that conventional methods can markedly underestimate the complexity of parasite populations [125]. This example underscores that the choice of diagnostic method is not merely a clinical question but a surveillance design decision with direct epidemiological consequences.

Addressing these interconnected challenges requires integrating molecular diagnostics, ecological surveillance, and cross-sector data—spanning human clinical records, veterinary findings, vector monitoring, and environmental information—within a coordinated One Health framework [139]. Such integration supports early detection of emerging threats, rational use of antiparasitic treatments, resistance monitoring, and the design of targeted interventions, including vector control and vaccination strategies.

Looking ahead, closing the diagnostic gap for vector-borne protozoal infections will require coordinated investment across several fronts: development and validation of standardised, accessible molecular tools for neglected livestock pathogens; expansion of cross-sector surveillance networks that link veterinary, wildlife, and human health data; and greater harmonisation of reporting frameworks at the national and international levels. Advances in portable sequencing, multiplex isothermal amplification, and AI-assisted image analysis hold genuine promise for field-deployable diagnostics in resource-limited settings. Realising this potential will depend not only on technological development but also on political and institutional commitment to One Health as an operational—rather than merely aspirational—principle.

## 6. Conclusions

The framework presented in this review is best illustrated through concrete examples. *Trypanosoma equiperdum* and *T. evansi* are closely related at the genome level, and their trypomastigote stages are morphologically indistinguishable; what separates them in practice is where in the host the parasite is found—*T. equiperdum* is predominantly localised to genital and neural tissues, *T. evansi* is typically detectable in peripheral blood, especially during active parasitaemia—making sample selection (genital swab vs. blood) the primary diagnostic decision and serology a particular challenge given the antigenic overlap. Likewise, *Leishmania* amastigotes and intracellular *Trypanosoma cruzi* amastigote nests can be confused on tissue smears or histology, with the differential resting on host species, geographic origin, lesion location, and tissue tropism (cutaneous vs. cardiac/intestinal); molecular confirmation is often decisive. These examples make concrete the dependencies—between host species, anatomical site, parasite biology, and assay choice—that the following priorities are designed to address.

Building on this framework, four priorities emerge for advancing veterinary diagnosis of vector-borne protozoal infections:

1. Validated, regionally appropriate molecular assays. Routine clinical use of well-characterised PCR and qPCR assays—with documented analytical and clinical performance in the target region—would substantially improve diagnostic accuracy beyond what serology alone can achieve, particularly for active infection, treatment monitoring, and discrimination of closely related parasites, and especially for parasites with substantial geographic genetic diversity—such as *T. cruzi* DTUs, *Leishmania* species complexes, and *Babesia*/*Theileria* genotypes—where regional variation may affect primer binding, assay sensitivity, and specificity. Where vaccination is in use (notably canine leishmaniosis), molecular confirmation has become essential for distinguishing infection from vaccine-induced serology.

2. One Health integration. Companion animals (especially dogs) are sentinels and reservoirs for several zoonotic vector-borne protozoa, including *T. cruzi* and *Leishmania* spp. Integrated veterinary–human surveillance—sharing diagnostic platforms, sample banks, and reporting frameworks—would strengthen early detection of geographic expansion, emerging genotypes, and spillover events. This integration is particularly important for parasites for which human commercial assays exist, but veterinary equivalents do not.

Equally important is the reverse flow of information: surveillance data on circulating species, genotypes, and resistance patterns should be made accessible to practising clinicians through regularly updated, openly available networks, so that test selection, interpretation, and case management reflect current local epidemiology rather than outdated regional assumptions.

3. Cross-geographical evaluation of diagnostic assays. As emphasised in Section 3.3, single-region validation of serological and molecular assays can yield performance estimates that do not generalise. Coordinated multi-site evaluation across endemic settings—covering the full range of circulating parasite genotypes, host species mixes, and transmission intensities—would provide more realistic field-performance estimates and reduce diagnostic mismatch when assays are deployed outside their original validation context.

4. Diagnostic strategies tailored to endemicity and resource setting. No single diagnostic configuration fits every veterinary setting. In high-endemicity regions with stable transmission, where seroprevalence baselines are high and a positive single-time-point result has limited specificity for active disease, surveillance design must prioritise molecular confirmation, paired-sera dynamics, or integrated clinical and exposure data over seroprevalence alone. Importantly, the optimal diagnostic configuration also differs according to whether the objective is clinical case confirmation, herd or population surveillance, or regulatory certification for trade.

In low-endemicity or outbreak-prone settings, by contrast, a positive serological result carries higher predictive value, but local laboratory expertise may be more limited; here, point-of-care lateral flow assays and dried-blood-spot strategies that can be processed centrally have a particular role, complemented by molecular confirmation at reference laboratories.

In resource-limited field settings, the practical case for AI-assisted microscopy depends on the availability of veterinary-relevant curated image repositories, smartphone-compatible acquisition workflows, and validated free-to-use algorithms—none of which has yet been validated and deployed at the scale required for routine veterinary protozoal diagnostics, and all of which warrant coordinated investment.

A specific challenge that will intensify in the next decade is the surveillance interference introduced by canine leishmaniosis vaccination. As vaccination coverage rises in endemic Mediterranean and Latin American regions, traditional whole-antigen serological surveillance becomes progressively less informative, and the field will increasingly need to rely on molecular surveillance, recombinant-antigen serology validated against vaccine cross-reactivity, or DIVA-compatible vaccines whose use does not interfere with infection diagnosis.

Accurate diagnosis of vector-borne protozoal infections in veterinary medicine requires an integrated approach that matches the selected method to the biological characteristics of the suspected pathogen, the clinical context, and the available resources. Microscopy provides rapid, direct parasite identification but demands expertise and is not sensitive enough for low-parasitaemia infections or for species such as *T. equiperdum* and *Leishmania* spp. that do not circulate in peripheral blood. Serological methods, for which more commercially available kits exist, extend diagnostic reach to subclinical and chronic infections and are indispensable for population screening and trade certification, but cannot distinguish active infection from prior exposure and are hampered by cross-reactivity among related taxa. Molecular diagnostics offer the highest sensitivity and resolution, enabling species- and genotype-level discrimination and quantitative estimation of parasite burden, yet their availability in veterinary settings remains severely limited—particularly for livestock trypanosomoses and emerging piroplasmid variants. Target selection critically determines assay performance: short 18S rRNA amplicons are often sufficient for broad screening or genus-level detection but may lack discriminatory power for closely related genotypes in Piroplasmida, where longer fragments or protein-coding gene targets are required. Although many protocols have proved specific and sensitive, few are commercially available; their adoption by reference laboratories and veterinary surveillance consortia would nevertheless be invaluable. Placing these diagnostic considerations within a One Health framework is not merely aspirational—it is operationally necessary. Animals serve as reservoirs, sentinels, and amplifying hosts for many of the pathogens reviewed here, and the fragmentation of veterinary surveillance substantially limits our capacity for early detection of zoonotic threats.

Coordinated investment in accessible, validated molecular tools, cross-sector data integration, and harmonised reporting standards is essential to close the diagnostic gap that currently impedes both animal health management and public health preparedness.

Ultimately, even the best-validated assays and most thoughtfully designed surveillance frameworks will remain wishful thinking unless national and regional authorities translate them into action. This requires three concrete commitments from local governments: incorporating evidence-based diagnostic and surveillance recommendations into national animal-health policy and veterinary regulatory frameworks; allocating sustained funding for laboratory infrastructure, reagent supply chains, personnel training, and field implementation; and establishing—or strengthening—mandatory reporting systems that capture veterinary case and surveillance data domestically and transmit it transparently to international bodies such as the World Organisation for Animal Health (WOAH) and the FAO. Without political commitment, dedicated investment, and a functioning reporting architecture that connects the practising veterinarian to the international surveillance system, the diagnostic advances reviewed here will deliver only a fraction of their potential public- and animal-health value.

## Figures and Tables

**Table 1:** Published AI-assisted microscopy algorithms for vector-borne protozoal parasites of veterinary relevance.

Host	Parasite(s)	AI Method *	Accuracy (%)	Precision (%)	Recall (%)	F1-Score (%)	Reference	Dataset Size **
Dog	*Babesia* spp.	WeightedEnsemble	97.75	98	97.5	98	[36]	4000
Dog	*Babesia gibsoni*	SimCLR (EfficientNet_b2)	97.09	94.5	97.3	95.9	[37]	1578
Horse	*Theileria equi*, *Babesia caballi*	YOLOv8 ‡	91	98	92	95	[38] ‡	2000
Poultry	*Plasmodium gallinaceum*	YOLOv3 + Darknet	99.2	— §	99.2	—	[39]	12,761
Mice	*Trypanosoma cruzi*	ML—Random Forest	89.5	87.6	—	89	[40]	2628
Human/Animal (archived)	*Trypanosoma brucei*, *T. cruzi*, *Trypanosoma evansi*	Deep Metric Learning (ResNet50 + CBIR/KNN)	99.71	93.5	96.6	94.9	[41]	32,276
Multiple (archived)	*Babesia* spp., *Leishmania* spp., *Plasmodium* spp., *Trypanosoma* spp.	BYOL SSL (ResNet50)	99.2	98.9	98.2	98.7	[42]	33,694

* When various models were tested, the model with the best results was included. ** Total number of images used for training and evaluation (test and controls). ‡ Article in Indonesian; title and content translated using Google Translate. § The paper reports specificity (≥99%), not precision; these metrics are not equivalent, and the value has been omitted.

## Data Availability

No new data were created or analysed in this study.

## Supplementary Materials

The following supporting information can be downloaded at: https://www.mdpi.com/article/10.3390/pathogens15060561/s1. Table S1: Distribution of vector-borne protozoal parasites of veterinary relevance by host species and geographic region; Table S2: Commercial diagnostic tests for vector-borne protozoal parasites of veterinary importance—Sheet 1: serological assays, Sheet 2: commercial molecular diagnostic kits; Table S3: Published primer sets and molecular assay designs for veterinary vector-borne protozoa, including target genes, amplification methods, and detection formats.

## Abbreviations

The following abbreviations are used in this manuscript:

AI	artificial intelligence
BYOL	bootstrap your own latent (self-supervised learning method)
CATT	card agglutination test for trypanosomiasis
CBIR	content-based image retrieval
CE-IVD	Conformité Européenne—In Vitro Diagnostic
cox3	cytochrome c oxidase subunit III gene
CRISPR	clustered regularly interspaced short palindromic repeats
ddPCR	droplet digital PCR
DAT	direct agglutination test
DIVA	Differentiating Infected from Vaccinated Animals
DNA	deoxyribonucleic acid
DTU	discrete typing unit
ELISA	enzyme-linked immunosorbent assay
EMPRES-i+	Emergency Prevention System for Animal Health Global Information System
ERA-Cas12a	extension of recombinase-aided amplification coupled with CRISPR-Cas12a detection
FAO	Food and Agriculture Organization of the United Nations
FDA	U.S. Food and Drug Administration
FIND	Foundation for Innovative New Diagnostics
HAT	human African trypanosomiasis
IFAT	indirect fluorescence antibody test
ITS	internal transcribed spacer
kDNA	kinetoplast DNA
KNN	k-nearest neighbours
LAMP	loop-mediated isothermal amplification
LDT	laboratory-developed test
mAECT	miniature anion-exchange centrifugation technique
ML	machine learning
MPSP	major piroplasm surface protein
mRNA	messenger RNA
nad5	NADH dehydrogenase subunit 5 gene
NCBI	National Center for Biotechnology Information
nMLST	nuclear multilocus sequence typing
nUPDx	nested universal parasite diagnostic assay
PAAT	Programme Against African Trypanosomosis
PAHO	Pan American Health Organization
PCR	polymerase chain reaction
POC	point of care
QBC	quantitative buffy coat
qPCR	quantitative PCR
rDNA	ribosomal DNA
RDT	rapid diagnostic test
RNA	ribonucleic acid
RUO	Research Use Only
RPA	recombinase polymerase amplification
rRNA	ribosomal RNA
SAR	Stramenopiles–Alveolates–Rhizaria (a major eukaryotic supergroup)
SSL	self-supervised learning
SSU	small subunit (ribosomal RNA)
SYBR	SYBR Green intercalating fluorescent dye
VEuMAP	VEuPathDB map (eukaryotic pathogen epidemiological and population-level data platform)
WAHIS	World Animal Health Information System
WHO	World Health Organization
WOAH	World Organisation for Animal Health
